# Molecular tools in understanding the evolution of *Vibrio cholerae*

**DOI:** 10.3389/fmicb.2015.01040

**Published:** 2015-10-06

**Authors:** Md. Habibur Rahaman, Tarequl Islam, Rita R. Colwell, Munirul Alam

**Affiliations:** ^1^Department of Biology and Chemistry, North South University, DhakaBangladesh; ^2^Enteric and Food Microbiology Lab, Center for Communicable Diseases, International Center for Diarrheal Disease Research, DhakaBangladesh; ^3^Maryland Pathogen Research Institute, University of Maryland, College Park, MDUSA; ^4^Center for Bioinformatics and Computational Biology, University of Maryland, College Park, MDUSA

**Keywords:** cholera, *V. cholerae*, molecular fingerprinting, PFGE, MLST, MLVA, whole genome sequencing

## Abstract

*Vibrio cholerae*, the etiological agent of cholera, has been a scourge for centuries. Cholera remains a serious health threat for developing countries and has been responsible for millions of deaths globally over the past 200 years. Identification of *V. cholerae* has been accomplished using a variety of methods, ranging from phenotypic strategies to DNA based molecular typing and currently whole genomic approaches. This array of methods has been adopted in epidemiological investigations, either singly or in the aggregate, and more recently for evolutionary analyses of *V. cholerae*. Because the new technologies have been developed at an ever increasing pace, this review of the range of fingerprinting strategies, their relative advantages and limitations, and cholera case studies was undertaken. The task was challenging, considering the vast amount of the information available. To assist the study, key references representative of several areas of research are provided with the intent to provide readers with a comprehensive view of recent advances in the molecular epidemiology of *V. cholerae.* Suggestions for ways to obviate many of the current limitations of typing techniques are also provided. In summary, a comparative report has been prepared that includes the range from traditional typing to whole genomic strategies.

## Introduction

Cholera is a severe and watery form of diarrhea caused by the pathogen, *Vibrio cholerae.* Clinical manifestations range from voluminous stool, hypo-volemic shock, to acidosis (Kaper et al., 1995). Severe fluid loss can lead to death within a day of onset (Sack et al., 2004). To date, seven pandemic outbreaks of cholera have been classified by year of commencement, namely 1817, 1829, 1852, 1861, 1881, 1899, and 1961 (Stine and Morris, 2014). The current seventh pandemic is believed to have originated in Indonesia (Barua, 1972) and continued in other continents, typical of pandemics. The current cholera pandemic is reported to be the most extensive, in terms of duration and geography (Faruque and Mekalanos, 2012). Earlier pandemics were speculated to have originated in the Ganges Delta region of the Indian subcontinent (Singh and Mohapatra, 2008). In addition to recorded pandemics, many localized outbreaks have also affected regions with severe outcomes, as occurred with re-emergence of cholera in Latin America in 1991 and explosive outbreaks of cholera in Orissa 1999, Dhaka 2006, Zimbabwe 2008, Haiti 2010, and Kenya 2010 (Singh and Mohapatra, 2008; Stine and Morris, 2014). All of these events highlight the epidemiological importance of this global disease. Ranges of typing and whole genomic approaches have been adopted for effective tracking of the evolution of *V. cholerae*, an overview of which is provided here, showing the overall impact on the changing paradigm of *V. cholerae* epidemiology and evolution.

## Cholera Epidemiology

**FIGURE 1:**
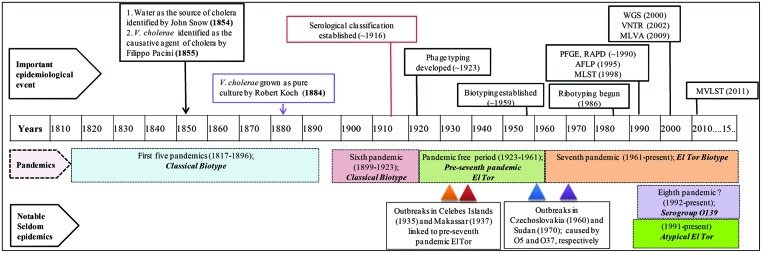
**Timeline of cholera epidemiology since 1817.** The upper panel shows important scientific advances that have changed the landscape of cholera epidemiology. Pandemics and their putative causative strains are shown in shaded boxes with dashed boarders. The lowermost panels show infrequent outbreaks and their causative strains, with triangles indicating time of outbreaks. This timeline has been adapted from Banerjee et al. (2014). PFGE, pulsed-field gel electrophoresis; RAPD, random amplification of polymorphic DNA; AFLP, amplified fragment length polymorphism; MLST, multi-locus sequence typing; WGS, whole genome sequencing; VNTRs, variable number of tandem repeats; MLVA, multi-locus variable tandem repeat; and MVLST, multi-virulence locus sequencing typing.

Cholera, a curse for centuries, is speculated to have originated before the time of Hippocrates and Buddha (Barua, 1992). However, it was not until 1854, when the classical epidemiological study of John Snow mapped the waterborne transmission of cholera in London (Snow, 1855), even before the notion of microbial causation of cholera. Subsequently, Filippo Pacini identified *V. cholerae* as the causative agent of cholera in 1855 (Bentivoglio and Pacini, 1995), a finding that was overshadowed when Robert Koch was able to grow the “*Vibrio comma”* in culture in 1884 (Koch, 1884; **Figure 1**). All of these findings overruled many of the misguided notions about cholera, as it was once thought that cholera was spread by “miasma” or “fog of rivers” (Sack et al., 2004).

During the first six cholera pandemics, *V. cholerae* classical biotype was isolated globally between 1817 and 1923 (Blake, 1994; Dziejman et al., 2002). Based on historical records, the period between 1923 and 1961 was pandemic free, although a major epidemic outbreak was recorded in the Celebes Islands (currently Indonesia) in 1935 caused by a new biotype, ‘El Tor’ (*V. cholerae MAK757*). The ‘El Tor’ biotype was also isolated in Makassar, Indonesia (strain M66-2) during a cholera outbreak in 1937. Both outbreaks were not pandemics and de Moor described them as ‘Paracholera’ and van Loghem as ‘Enteritis choleriformis El Tor,’ but the *MAK757* and M66-2 isolates were subsequently labeled as pre-seventh pandemic El Tor (Barua, 1992; Banerjee et al., 2014; **Figure 1**).

The seventh cholera pandemic is reported to have been caused by the El Tor biotype that gradually replaced classical strains. This was the only pandemic that had originated outside of India, namely in Sulawesi, Indonesia, in 1961 and was isolated from cholera patients in territories of Asia by 1966. Until 1971, cholera outbreaks were few, while in Africa and Europe, an upsurge was recorded and until then, cholera had not been reported in those countries for more than 100 years (Reeves and Lan, 1998). After a lull, El Tor again caused a massive outbreak in 1991 in Peru, the first cholera epidemic in Latin America since 1895 (Seas et al., 2000). In 1992, a new variant of the seventh pandemic strain appeared in Madras and spread rapidly in Asia, raising concerns about the beginning of an eighth pandemic of cholera (Siddique et al., 1996; **Figure 1**). This new pathogenic serotype is referred to as *V. cholerae* O139 Bengal, the O1 antigen of the prototypic seventh pandemic strain having been replaced by the O139 antigen, hence a new serogroup (Albert, 1994). Later, the emergence of new variants of *V. cholerae* O1 harboring traits of both classical and El Tor biotypes was recorded (Nair et al., 2002, 2006; Ansaruzzaman et al., 2004), collectively referred to as ‘atypical El Tor’ (Safa et al., 2010). The ongoing seventh pandemic is the longest of the cholera pandemics and is believed to continue because the causative agent, El Tor and its derivatives are presumed better adapted for global dissemination compared to their classical predecessors (Faruque et al., 1998; Safa et al., 2010). Although *V. cholerae* strains belonging to O1 and O139 serogroups have been responsible for both major epidemics and endemic cholera, other serogroups are referred to as *V. cholerae* non-O1/non-O139. They have also caused cholera, but rarely epidemics, an example of which occurred in the 1960 and 1970s, when *V. cholerae* O5 and O37 serogroups caused explosive outbreaks of cholera in Czechoslovakia and Sudan, respectively (Aldová et al., 1968; Kamal, 1971; Yamamoto et al., 1995; **Figure 1**).

Although seven cholera pandemics have been recorded since 1817, isolates from the first five pandemics are not available because *V. cholerae* was first isolated and identified only at the time of the fifth cholera pandemic (Koch, 1884). Evolution of diagnostic and fingerprinting strategies since then have enabled study of sixth and seventh pandemic strains in detail (Reeves and Lan, 1998). In this review, we have grouped fingerprinting methods into three categories, phenotypic/traditional, pre-genomic, and genomic, showing estimated time of development and the influence of each on cholera epidemiology (**Figure 1**).

## Phenotypic Fingerprinting

**FIGURE 2:**
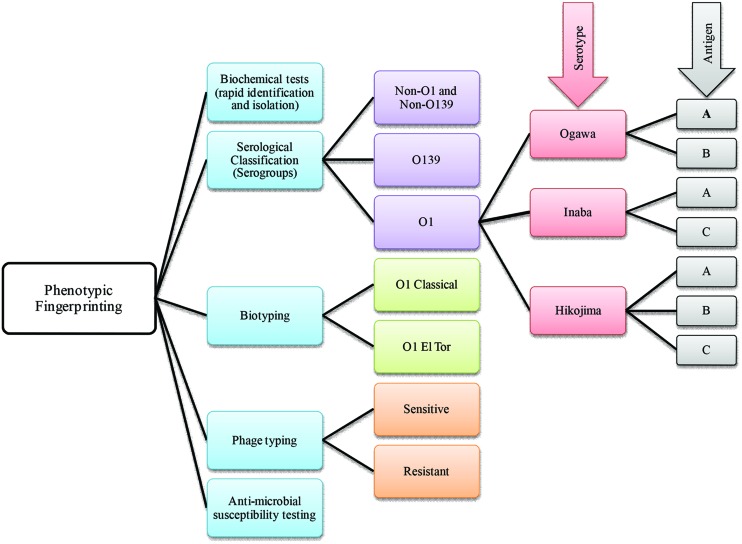
**A brief scheme that includes phenotype based fingerprinting strategies employed in the classification of *Vibrio cholerae***.

Phenotype based traditional typing methods, namely serotype, biotype, antibiogram, and phagetyping have been used for many years, although eventually challenged because of their relatively low discriminatory power (Bhowmick et al., 2011). Among phenotypic strategies, serological classification of cholera *vibrios* was established during the sixth cholera pandemic (Greig, 1916). Later, the most widely used typing scheme was developed by Sakazaki and Shimada (1977) who used anti-sera of heat killed microbes. The scheme identified 138 *V. cholerae* O serogroups, to which the O139 serogroup was added (Bhattacharya et al., 1993). In the USA, a similar scheme using anti-sera of live organisms was also used for serological classification of *V. cholerae* (Smith, 1979). All of these serological classifications are based on differences in the sugar composition of the heat-stable surface somatic ‘O’ antigen of *V. cholerae* (Gardner and Venkatraman, 1935). Moreover, based on antigenic factors, *V. cholerae* O1 has been differentiated into three serotypes, referred to as Ogawa, Inaba, and Hikojima (Banerjee et al., 2014; **Figure 2**).

Historically, two major biotypes of *V. cholerae*, Classical and El Tor, are recognized. El Tor biotypes are generally hemolytic, whereas classical biotypes are usually non-hemolytic (Pollitzer et al., 1959). Since exceptions to every rule may be found in biology sooner or later, it is not surprising that most El Tor biotype strains are non-hemolytic except isolates obtained in the early 1960s (Barrett and Blake, 1981). Therefore, hemolysis biotyping has limited utility because of inconsistency. The hemeagglutinating property of *vibrios* was explored by Doorenbos in 1932 (Felsenfeld, 1966), and later applied as a biotyping tool, using chicken red blood cells which agglutinate with El Tor but not with classical *V. cholerae* (Finkelstein and Mukerjee, 1963). In addition, the Voges–Proskauer test, resistance to polymyxin B (Han and Khie, 1963), and modified CAMP test (Lesmana et al., 1994) have been used for determining *V. cholerae* biotypes.

*Vibrio cholerae* belonging to the same serotype or biotype can be further differentiated by phage typing. One of the earlier studies on cholera phages was carried out by Nobechi in 1923 and has been summarized (Pollitzer et al., 1959). Subsequently, phage typing was done using Mukerjee’s cholera phages (Basu and Mukerjee, 1968). The FK phages of Takeya et al. (1981) later were adopted to differentiate classical and El Tor biotypes. In both cases, phage susceptibility was used as a method for strain differentiation. Mukerjee’s phage typing scheme was efficient when used to study the initial spread of *V. cholerae* O1 El Tor (Faruque, 2014) also, phage typing has proven useful when phenotypic fingerprinting was inconclusive. Over the course of time, phage typing schemes proved inadequate for typing the large number of emerging *V. cholerae* strains. The fact that the number of well characterized *Vibrio* phages was limited and consensus for typing schemes was not uniform, posed major challenges to the use of phage sensitivity and resistance as a major typing tool (Kaper et al., 1995). Interestingly, new phage typing schemes for *V. cholerae* O1 El Tor (Chattopadhyay et al., 1993) and O139 strains (Chakrabarti et al., 2000) have been developed that appear to overcome some of the limitations.

Antimicrobial susceptibility has been used to characterize *Vibrio* spp. and their susceptibility to various antibiotics is measured by using a battery of antibiotics at various concentrations. The resistances are scored to generate a fingerprint, i.e., an antibiotic resistance profile, which is subjected to cluster analysis, and comparison to a reference database for microbial tracking (Scott et al., 2002). Changing antibiotic resistance patterns has been described as a hallmark of cholera epidemiology and is associated with the substantial mobility of genetic elements harboring antibiotic resistance genes in *V. cholerae* (Faruque et al., 1998). In addition to the standard disk diffusion method recommended by the Clinical Laboratory and Standard Institute (CLSI), a rapid Vitek susceptibility system has been developed to determine antibiotic resistance profiles of *V. cholerae* O1, O139, and Non-O1 (Sciortino et al., 1996).

Although phenotypic fingerprinting strategies have been used routinely for identification and tracking of *V. cholerae* since the beginning of the seventh pandemic, these tools have major disadvantages, including unstable phenotypes, lower sensitivity, and limited specificity.

## Pre-genomic Era of Fingerprinting

Relatedness and differences among bacterial isolates derived from molecular signatures at the DNA level have been used for molecular fingerprinting (Sabat et al., 2013). The applicability of a given fingerprinting method depends on its discriminatory power, that is, its ability to distinguish epidemiologically unrelated isolates and determine those closely related. In addition, speed, reproducibility, portability among laboratories, cost and ease of interpretation is major considerations (Struelens, 1996; van Belkum et al., 2007). Molecular methods have proven effective for determining genetic changes that result in displacement of an existing serogroup with a newly emerging serogroup.

**FIGURE 3:**
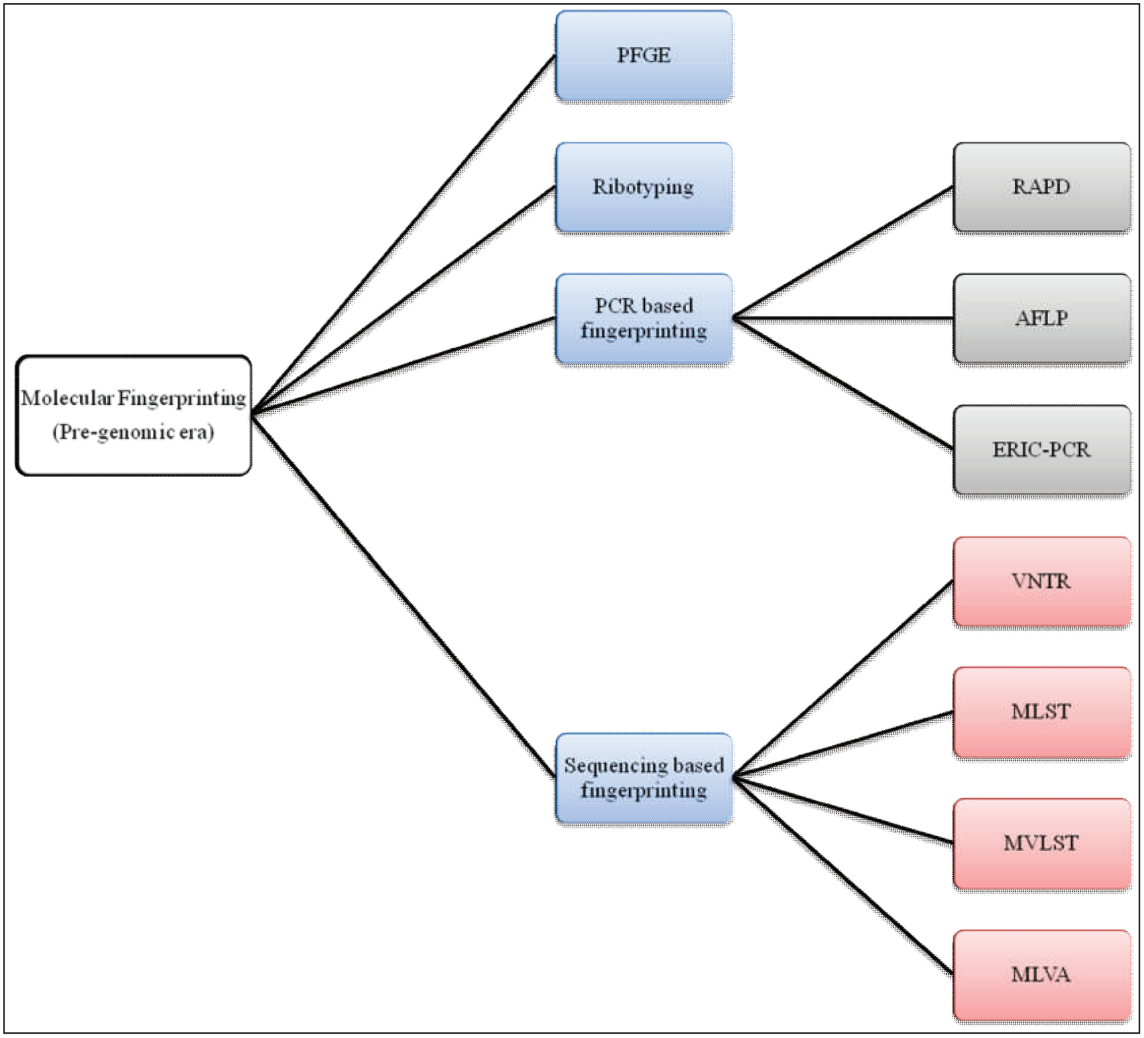
**A chart of molecular fingerprinting strategies used in epidemiological investigations of *V. cholerae***.

**FIGURE 4:**
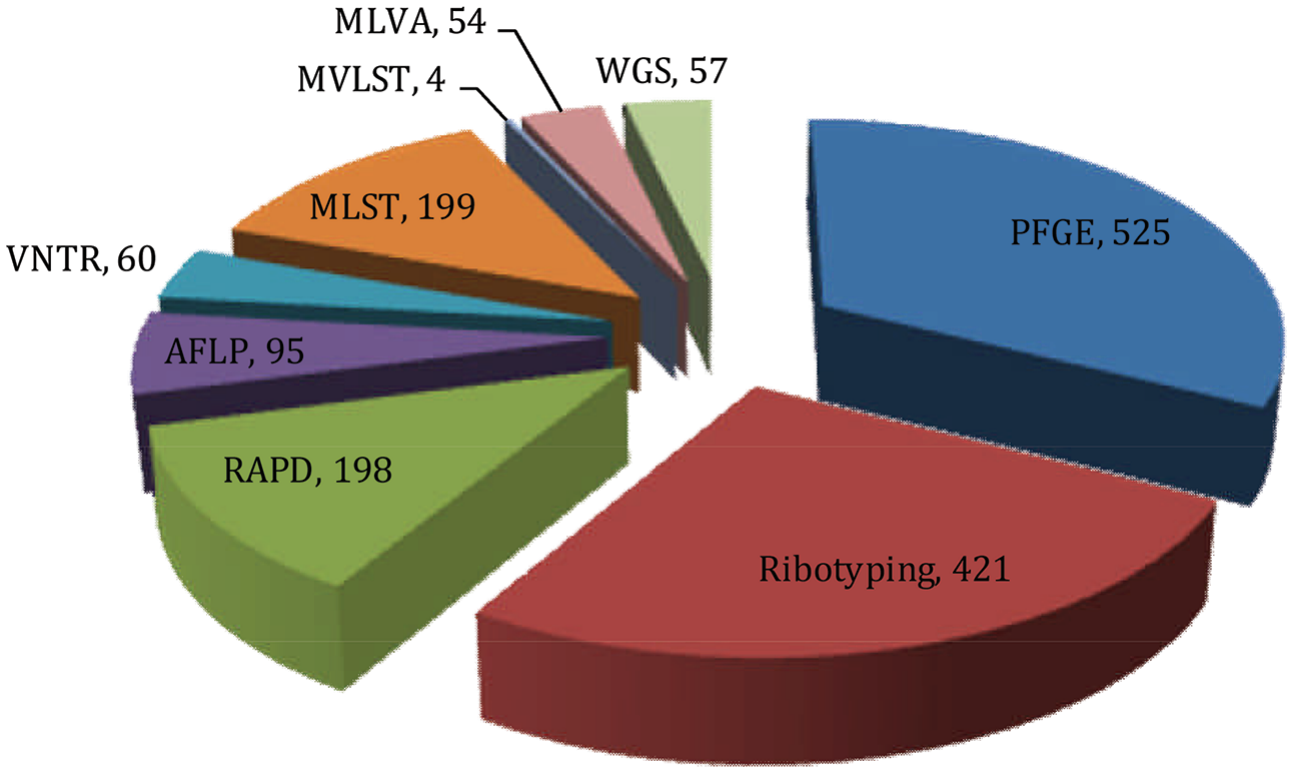
**Pie chart showing the number of publications retrieved from the literature database (HighWire and PubMed) that cited corresponding fingerprinting tools employed in the molecular epidemiology of *V. cholerae* (Data retrieved up to July, 2015)**.

Stine and Morris (2014) proposed acqisition of a novel genetic element(s) leads to an increase in bacterial fitness which, in turn, causes a selective sweep (a local or global outbreak). The seventh cholera pandemic was suggested to comprise four such selective sweeps. The first selective sweep is considered to be associated with emergence of *V. cholerae* O1 El Tor in 1961, whereby the ancestor of *V. cholerae* O1 El Tor strains acquired *Vibrio* seventh pandemic Islands (VSPs) I and II (Dziejman et al., 2002). The second selective sweep was considered to have begun with acquisation in 1981 of the novel sxt element. The sxt element harbors several antibiotic resistance genes that provide selective advantages to the host and is present in almost all post-1990 *V. cholerae* O1 clinical isolates (Waldor et al., 1996). The third selective sweep is considered to have been triggered by replacement of *V. cholerae* O1 serogroup encoding genes with those for O139, resulting in an outbreak of cholera across the Indian subcontinent (Bik et al., 1995; Comstock et al., 1996; Mooi and Bik, 1997). The fourth, or most recent selective sweep, is proposed to have been initiated by replacement of the El Tor ctx allele with the classical ctx allele in an El Tor background (Raychoudhuri et al., 2009), presumed to be associated with the acute form of cholera (Nair et al., 2002). Molecular fingerprinting has been used to elucidate all of these marker events in cholera epidemiology leading to a growing interest in its clinical applications. In fact, several DNA-based strategies have been used for molecular typing of *V. cholerae*, exemplified by pulsed-field gel electrophoresis (PFGE), ribotyping, random amplification of polymorphic DNA (RAPD), AFLP, enterobacterial repetitive intergenic consensus sequence-PCR (ERIC-PCR), VNTRs, multi-locus sequence typing (MLST), and multi-locus variable tandem repeat analysis (MLVA; Bhowmick et al., 2011; **Figure 3**). To underscore the impact of these methods on cholera epidemiology, publications that have cited molecular fingerprinting strategies are summarized as shown in **Figure 4**.

### Pulsed-Field Gel Electrophoresis

Among the various molecular fingerprinting methods, PFGE has proven most effective in epidemiological investigation of *V. cholerae* and is considered the ‘gold standard’ of typing (Tenover et al., 1997; Sabat et al., 2013). The method was used in epidemiological investigations of various bacterial species during the 1990s (Arbeit et al., 1990; Prévost et al., 1991; Gordillo et al., 1993; Tenover et al., 1995). Using the searching keyword ‘PFGE AND *V. cholerae*,’ 525 publications have been retrieved from the HighWire literature database^1^ in which PubMed^2^ was also included. The very large number of publications indicates the importance and influence of this method for molecular typing of *V. cholerae.*

Pulsed-field gel electrophoresis requires highly purified genomic DNA which is chopped with restriction endonuclease and the restriction fragments separated by applying alternating electric fields, i.e., a ‘pulsed-field,’ to obtain better resolution of separated fragments. Larger DNA fragments, with sizes ranging from 30 kb to 1 Mb, can be resolved using this typing approach (Goering, 2010). PFGE has been used frequently because of its high epidemiological concordance and excellent discriminatory power to distinguish among closely related isolates. Excellent typability, intra-laboratory reproducibility, and less expensive bench-top deployment provide added value (Sabat et al., 2013). However, the huge amount of PFGE-typing data generated over the last decade has challenged the scientific community to seek a common platform to improve inter-laboratory comparability. A robust, reproducible, and standardized PFGE protocol was sought (Cooper et al., 2006) and the networks, PulseNet (Swaminathan et al., 2001), and Harmony (Murchan et al., 2003), were formed.

An earlier study highlighted the usefulness of PFGE, notably in revealing clonality among isolates obtained from two well-defined cholera outbreaks in Malaysia. Restriction endonuclease analysis (REA) patterns of *V. cholerae* O1 El Tor strains from the two outbreaks were found to be similar but not identical. They varied widely from strains isolated from sporadic cases of cholera (Mahalingam et al., 1994). Cameron et al. (1994) undertook a comparative study that included PFGE, multi-locus enzyme electrophoresis (MEE) and ribotyping, using 180 isolates of *V. cholerae* O1. They concluded PFGE profiling was more discriminating for the *V. cholerae* O1 serogroup, compared to the other subtyping assays and recommended it as an effective tool for epidemiological surveillance (Cameron et al., 1994). PFGE based molecular typing provided new insight into the 1991 cholera epidemic in Mexico in a study carried out by Alam et al. (2012), who suggested there was a regional signature in the evolution of classical biotypes in the Americas, independent from the geographically ecosystem of Asia. Recently, PFGE analysis of Mexican isolates collected between 1998 and 2008 revealed a progression of CTX^+^ El Tor harboring truncated CTX prophage, the predominant cause of endemic cholera in Mexico despite altered El Tor (carrying classical CTX allele) being the predominant cholera causing agent worldwide. This was reported to be a key historical event in the global epidemiology of cholera and PFGE was concluded to be an effective fingerprinting tool (Alam et al., 2014).

Although, PFGE has been widely used in molecular epidemiology studies of *V. cholerae*, some of its drawbacks have to be taken into consideration. The method is technically demanding, labor-intensive, relatively slow, and has limited data portability, compared to sequence-based methods. In addition, inter-laboratory comparability of complex PFGE-patterns is technically challenging. Single mutations at cutting sites can result in altered restriction profiles and PFGE cannot distinguish among nearly identical band sizes differing by less than 5% (Sabat et al., 2013). Nonetheless, PFGE is used in combination with other typing strategies that compensate for many of its limitations. Teh et al. (2010) demonstrated that PFGE in combination with multi-locus VNTR (MLVA) can provide more information on epidemiological relatedness and differences among isolates from different sources or geographical regions.

### Ribotyping

In a search using the keywords ‘Ribotyping AND *V. cholerae*,’ 421 publications were retrieved from the literature database, indicating the significance of this method in defining the biogeography and molecular epidemiology of *V. cholerae*. Since the emergence of ribotyping in Grimont and Grimont (1986), many studies showed that the technique alone or in combination with other methods had been effective in studies of the molecular evolution of *V. cholerae* (Lan and Reeves, 1998; Pourshafie et al., 2000, 2002).

In general, four major consecutive steps are usually employed in ribotyping: (a) restriction digestion of the bacterial chromosome; (b) gel electrophoresis of the resulting fragments; (c) transfer of the fragments to a membrane and; (d) hybridization of the fragments with labeled probes complementary to the 16S and 23S rRNAs (Grimont and Grimont, 1986). However, wider interest led to adaptation of this procedure. To some extent, the name ‘ribotyping’ has been a misnomer. Based on *in silico* genomics, Bouchet et al. (2008) suggested that ribotypes are derived from restriction fragment length polymorphisms (RFLPs) of neutrally evolving housekeeping genes that flank rRNA genes, rather than directly from rRNA gene sequences that serve solely as conserved and linked tags. Using intergenic polymorphisms, i.e., the spaces between 16S and 23S rRNA genes, has also been adopted mainly in Europe for microbial fingerprinting, e.g., *Clostridium difficile* as described elsewhere (Sabat et al., 2013).

Popovic et al. (1993) proposed a standardized scheme for ribotyping used in epidemiological investigations of *V. cholerae*. Under their scheme, data collected during a 60 years follow up study were analyzed. A total of 214 *V. cholerae* O1 strains were isolated from 35 countries and 14 states of USA and grouped into 27 *BglI* ribotypes. Interestingly, it was found that strains causing the fifth, sixth, and ongoing seventh pandemic could be grouped into different ribotypes. Among the classical biotypes, seven different but similar ribotypic patterns (1a–1g) were observed, whereas, six different ribotypes with three subtypes were found among seventh pandemic strains. It was suggested that wide circulation of different clones favored persistence of *V. cholerae* in the environment but certain clones were restricted to specific regions, i.e., ribotype-5 was predominant in Latin America and ribotype-8 was restricted to central Africa (Popovic et al., 1993; Thompson et al., 2004). Later, Faruque et al. (1997) classified *V. cholerae* O1 El Tor strains into five ribotypes based on *BglI* restriction profiles. *V. cholerae* El Tor strains isolated before emergence of O139 were grouped as ‘ribotype I–IV’ and post-O139 isolates were grouped as ‘ribotype V’ (Faruque et al., 1997), which had not been described by Popovic et al. (1993).

An earlier ribotyping study had shown an association between clinical and environmental isolates of *V. cholerae* O1 during an 8 years follow-up study in Australia (Desmarchelier et al., 1995). A recent study undertaken in Bangladesh reported *V. cholerae* toxigenic O1 and O139 environmental strains shared similar ribotype profiles with pandemic strains. Furthermore, the *V. cholerae* non-O1, non-O139, and TCP**^-^** non-toxigenic O1 strains diverged widely from seventh pandemic O1 and O139 strains. Hence, it was suggested that there was heterogeneity among the environmental *V. cholerae* population in a cholera endemic area like Bangladesh (Faruque et al., 2004). Another earlier case study provided evidence that the presence of 11 ribotypic polymorphic restriction sites in seventh pandemic isolates can be used to distinguish them from sixth and pre-seventh pandemic isolates. However, ribotyping does not necessarily represent bacterial evolution because the pattern obtained may be the result of recombination events (Karaolis et al., 1994).

### Polymerase Chain Reaction (PCR) Fingerprinting

#### Random Amplification of Polymorphic DNA

Random amplification of polymorphic DNA is one of the earlier PCR based fingerprinting strategies which had been used to characterize and trace the phylogeny of diverse bacterial species of epidemiological importance. The technique was first developed in the 1990s by two independent groups (Welsh and McClelland, 1990; Williams et al., 1990). The search keywords ‘RAPD AND *V. cholerae’* was used to retrieve 198 publications from literature databases (as described above). These publications amplify the importance of this tool in studying *V. cholerae* molecular epidemiology. RAPD employs several arbitrary and short oligonucleotide primers (9–10 bases in length) to hybridize chromosomal DNA randomly at a lower annealing temperature. If two RAPD primers can hybridize in proper orientation and proximity (within a few kilo-base span); the fragments will be amplified, corresponding to the distance between the two primers. Resulting amplified fragments are resolved by agarose gel electrophoresis to generate a semi-unique profile of the RAPD reaction which, in theory, is characteristic of the particular bacterial isolates. Relationships between *V. cholerae* isolates can be determined by comparing their unique RAPD fingerprinting profiles. However, a standardized RAPD protocol has not been used to form a data bank for identification of *V. cholerae* O1 strains, as was studied in Brazil (Leal et al., 2004). Moreover, RAPD is sensitive to technical variations introduced by laboratory personnel, different DNA samples, and different sources of enzyme and primers. These can generate different banding patterns, hence poor reproducibility (Meunier and Grimont, 1993).

#### Amplified Fragment Length Polymorphism (AFLP)

Amplified fragment length polymorphism (AFLP) is another molecular fingerprinting approach that utilizes a subset of genomic fragments generated by restriction digestion. The technique was developed in Vos et al. (1995) and since then has been used for molecular typing of diverse bacterial species. From a literature database search using the keywords ‘AFLP AND *V. cholerae*,’ 95 publications were retrieved, highlighting the impact of this method for *V. cholerae* molecular epidemiology and evolution. In the AFLP method, genomic DNA is cleaved with two restriction enzymes and the sticky ends of the restriction fragments are ligated with double stranded adaptors. Subsequently, restriction fragments are selectively amplified by PCR, wherein the flanking adaptor sequences are used as the primer binding sites. Initial cycles of PCR are run with stringency to ensure fidelity of the reaction or to avoid non-specific amplification. The amplified fragments are resolved by an automated DNA sequencer and the resulting banding patterns used to decipher genetic relatedness among bacterial isolates (Mortimer and Arnold, 2001). An exemplar study used AFLP based typing to uncover genetic relatedness among environmental and clinical *V. cholerae* isolates and concluded that pathogenic strains may have arisen from non-toxigenic strains in the aquatic environment (Jiang et al., 2000). Another study reported AFLP can be used to decipher variation among seventh pandemic clones more effectively, compared to ribotyping. Furthermore, a temporal pattern of change was found among clones of *V. cholerae* in which strains isolated between the 1960 and 1970s clustered distinctly, when compared with isolates collected between the 1980 and 1990s (Lan and Reeves, 2002).

### Sequencing based Fingerprinting

#### Variable Number of Tandem Repeats Analysis

Many pathogenic *V. cholerae* O1 and O139 isolates demonstrate similar genetic profiles by PFGE (Basu et al., 2000), *CTX*-genotyping (Basu et al., 2000; Mukhopadhyay et al., 2001), and ribotyping (Faruque et al., 2000). Sequencing based genotypic approaches subsequently have been adopted to obviate the limitations of these methods. Among those strategies, variable number of tandem repeats (VNTRs) or simple sequence repeats (SSRs) provide a source of high genomic polymorphism to distinguish rigorously within and among *V. cholerae* isolates. Literature database searches, using the keywords ‘VNTR and *V. cholerae’* retrieved 60 publications showing a growing interest in VNTR as a fingerprinting tool.

Variable number of tandem repeats is short DNA sequence motifs that are repeated in tandem at a specific locus and represent a distinctive hereditary feature of an individual isolate. The first report of a VNTR locus in *V. cholerae* was published in 2002, in which the locus was designated *VcA*, located on chromosome 2, and consisting of a TGCTGT repeat (Vodop’ianov et al., 2002). The usefulness of *VcA* VNTR was further tested and the conclusion was that the discriminatory ability of *VcA* VNTR was better than PFGE (Bhowmick et al., 2010). Another study identified 17 VNTR loci that could be used to differentiate *V. cholerae* isolates not discriminated by PFGE. Two of the loci were reported to be stable during serial passage under specific culture conditions (Danin-Poleg et al., 2007). VNTRs have also been shown to be effective in assessing genetic relatedness among outbreak isolates from geographic co-located Bangladeshi villages and within a short time frame (Stine et al., 2008). The spread of specific genotypes of *V. cholerae* O1 and O139 was tracked spatiotemporally across another cholera endemic country, namely India, by exploiting VNTR as an effective fingerprinting tool (Ghosh et al., 2008).

#### Multi-locus Sequence Typing

Multi-locus sequence typing is a sequencing based fingerprinting tool of particular interest since its development in Maiden et al. (1998). It provides sequence-based resolution, is informational, and is technically feasible. Literature database search using the keywords ‘MLST AND *V. cholerae’* retrieved 199 publications showing applications of MLST as a fingerprinting tool. *V. cholerae* shows variation in three housekeeping genes, *gyrB, pgm*, and *recA*, presumed to be highly conserved. Two earlier studies had used MLST based molecular typing to analyze evolutionary relationships among *V. cholerae* clones isolated from different geographic regions (Byun et al., 1999; Farfán et al., 2002). Kotetishvili et al. (2003) used MLST, along with PFGE, to characterize 22 *V. cholerae* isolates representing epidemic and non-epidemic serogroups and concluded MLST to be more discriminatory than PFGE. Another study used MLST of nine genetic loci and found Mozambique isolates shared the same unique sequence type (ST) as *V. cholerae* O1 El Tor strain N16961, a seventh pandemic isolate (Lee et al., 2006). Along with utilizing housekeeping genes, a derivative of the MLST scheme was developed that is based entirely on virulence genes and has been coined multi-virulence locus sequencing typing (MVLST). A recent study showed MVLST is more discriminatory than traditional MLST, because MVLST differentiates outbreak strains and toxigenic from non-toxigenic subtypes. In combination, they proved to be more discriminatory and informative in studying local epidemiology of *V. cholerae* (Teh et al., 2011).

#### Multi-locus Variable Tandem Repeat Analysis

Multi-locus variable tandem repeat analysis is a sequencing based typing tool that targets five or six loci in the *V. cholerae* genome. Each locus harbors a tandem repeat of six or seven nucleotides which can be repeated 4–31 times. The length of the tandem repeats represents allele numbers in the corresponding locus, which in turn is used to infer a five-digit genotype, i.e., for the five different loci. Strains derived from the same ancestor are presumed to be part of a clonal complex and, therefore, presumed to differ only at a single locus in a five-digit genotype. In contrast, genotypes that differ at two or more loci are considered clonally unrelated, hence evolved from different ancestors (Stine and Morris, 2014). A literature database search using the keywords ‘MLVA AND *V. cholerae’* retrieved 54 publications where MLVA was used as a fingerprinting tool.

One MLVA study showed the presence of five different clonal complexes in a set of Kenyan isolates (Mohamed et al., 2012) which was contrary to the hypothesis of a clonal introduction of outbreak causing strains (Stine and Morris, 2014). A recent study in Bangladesh exploited MLVA to determine clonal relationship between environmental and clinical *V. cholerae* O1 isolates from outbreaks in two geographically distinct locations. The study shed light on the mechanism of an accelerated mode of cholera transmission, i.e., person-to-person transmission, compared to slow mode transmission, namely the person-to-aquatic environment-to-person pathway (Rashed et al., 2014). Lam et al. (2012) showed that MLVA can be effective in resolution of closely related seventh pandemic clones which had been grouped on the basis of SNP profiles. An earlier study had developed a simple and highly discriminating MLVA strategy for connecting clinical and environmental *V. cholerae* isolates, using 12 VNTR loci among which six were found to be polymorphic. For those six loci, a higher discriminatory power (Simpsons Diversity Index = 0.99) was calculated for 142 environmental and clinical *V. cholerae* strains isolated from diverse geographic regions. MLVA was documented to be a potential fingerprinting tool for tracking the source of *V. cholerae* (Olsen et al., 2009).

## Fingerprinting in the Era of Genomics

Although molecular fingerprinting has been used for many years to study the molecular epidemiology of *V. cholerae*, thoroughness and resolution of these methods are limited. For instance, it was not until the late 1990s that the presence of two chromosomes in *Vibrio* species had been described by physical mapping (Trucksis et al., 1998; Yamaichi et al., 1999). The genomic era was launched by whole genome sequencing (WGS) of *V. cholerae* O1 El Tor N16961 by the joint consortium of The Institute for Genomic Research, the University of Maryland, and Harvard Medical School (Heidelberg et al., 2000). Functional annotation of the genome sequences and holistic resolution at the nucleotide level eased the way to high-throughput assays. Microarrays (Dziejman et al., 2002), parallel WGS (Grim et al., 2010; Mutreja et al., 2011), and hybrid *de novo* assembly of second and third generation sequencing data (Bashir et al., 2012) have since been used to redefine the intricate mechanisms of cholera pathogenesis and corollary outbreaks of the disease. A significant number of reports of whole genome based molecular epidemiology of *V. cholerae* indicate the field is growing rapidly.

After the initial WGS of *V. cholerae* by Heidelberg et al. (2000), comparative genomic analysis was conducted using classical, pre-pandemic, and pandemic El Tor, and two non-toxigenic *V. cholerae* N16961 strains in which a whole genome microarray of a seventh pandemic strain was used as a reference (Dziejman et al., 2002). All strains had shown remarkable similarity in gene content and each strain harbored at least 99% of the genes present in the reference strain. The *V. cholerae* O139 strains were found to be similar to the *V. cholerae* O1 El Tor strains and very likely were clonally derived from El Tor after a change in LPS. A seventh pandemic island was found only in the pandemic El Tor and O139 strains, but not in the classical or pre-pandemic El Tor strains. Genes encoded in the seventh pandemic island may function in persistence in the environment, hence a potential mechanism for replacement of classical strains by El Tor strains. These genes may allow seventh pandemic strains to withstand nutrient depletion or other environmental stresses or may be involved in interactions with non-human aquatic hosts (Chun et al., 2009).

Availability of extensive microbial genomic strain sequences led to the coining of a new terminology, the ‘pan-genome,’ comprising the core genome (indispensable genes shared by all strains) and the dispensable genome, i.e., genes not shared by all strains or genes that are unique to an individual strain (Tettelin et al., 2005). Both core and pan-genome genes are taken into consideration to deduce bacterial phylogenies. The core genome is more informative for determining phylogenetic relationships than ribotyping which exploits only variations in housekeeping genes flanking 16S/23S rRNA genes (Robins and Mekalanos, 2014). Chun et al. (2009) did a comparative genomic analysis of 23 *V. cholerae* strains and identified 2,432 common core genes, or orthologs, and 6,953 total unique genes in their pan-genome.

In the post-genomic era, second-generation (or ‘next-generation’) sequencing has been used extensively over the last decade for bacterial WGS, though their shorter read lengths remain as a major limitation. Next-generation sequencing merely produces draft or incomplete genomes, their higher contig numbers and varying qualities pose major challenges in genome assembly and closure (Land et al., 2014). As a result, majority of the currently available *Vibrio* genome sequences are reported to be incomplete draft sequences (Chapman et al., 2015). In contrary, the third generation (or ‘single molecule’) sequencing offers longer read lengths and potentially produces a finished or complete genome within a few hours. One of the first ‘single molecule’ sequencing platforms was whole genome optical mapping (Schwartz et al., 1993), in which the complete genomic map is deduced from a number of partial restriction maps, thus obviating the need for a physical map. It has proven effective for capturing and validating genomic complexities to produce a finished or complete genome (Niedringhaus et al., 2011). However, the dearth of standard statistical package and bioinformatic tools to analyze the myriad of sequencing data, sensitivity to stochastic insertion and deletion (indels) mutations, lower throughput and relatively higher expenses are among the major challenges for the routine application of the third generation sequencing, till now (Land et al., 2015).

The finished or complete genome is the ultimate target as it can aid in the deployment of *in silico* genomics precisely by using robust genome relatedness indices, e.g., average nucleotide identity (ANI; Konstantinidis et al., 2006). ANI values can potentially define the identity or similarity between two bacterial genomes by calculating the proportion of DNA shared by the genomes, and have been proposed as a ‘next-generation gold standard’ for species demarcation in the post-genomic era (Kim et al., 2014). ANI values coupled with comparative genomics have already been in use for identifying the close relatives of *V. cholerae* from environmental and clinical samples (Haley et al., 2010; Hasan et al., 2010; Kirchberger et al., 2014). Although the finished genome of *V. cholerae* is still in infancy, it has the potential to complement many of the traditional fingerprinting tools namely MLST and Ribotyping. For example, instead of typing 16S and 23S rRNA operons or other housekeeping genes such as *gyrB, pgm*, and *recA* by sequencing, those can be mapped directly from the complete genome reads to decipher variations related to the molecular evolution of *V. cholerae* (Kwong et al., 2015).

### Genomics and Haitian Cholera: A Case Study

Genomic tools potentiated an exploration of the mode of emergence and transmission of *V. cholerae* in the Haitian cholera epidemic that erupted in 2010 (Chin et al., 2011). This was the first whole genome study and showed strains of *V. cholerae* isolated during the Haitian cholera epidemic were related to South Asian seventh-pandemic O1 El Tor strains. It was hypothesized that these strains may have been introduced by human activities. Haitian strains were subsequently shown to be distinct from Latin American and East African strains of *V. cholerae* (Chin et al., 2011). The hypothesis of human transmission of a clonal strain from the South Asian region was supported by other investigators (Ali et al., 2011; Hendriksen et al., 2011; Reimer et al., 2011). Whole genome sequence typing (WGST) of Haitian and Nepalese strains indicated that they were related. Hendriksen et al. (2011) concluded that the 2010 cholera epidemic was initiated by Nepalese peacekeepers. However, based on whole genomic phylogeny and core genome SNPs, Reimer et al. (2011) concluded that the Haitian strain of *V. cholerae* O1 was genetically related to Indian and Cameroon strains. Chin et al. (2011) further concluded that Haitian cholera isolates could have originated from South Asia, not Nepal.

Hasan et al. (2012) showed both Haitian *V. cholerae* O1 and *V. cholerae* non-O1/O139 isolates had been isolated from Haitian cholera patient samples, based on WGS. Furthermore, they found non-O1/O139 strains were the sole pathogen in 21% of clinical specimens that had been analyzed (Hasan et al., 2012). They suggested autochthonous *V. cholerae* non-O1/O139 may serve as a reservoir for horizontally transmitted genes, as well as being pathogenic as has been reported elsewhere (Haley et al., 2012). In addition, a hydro-climatological study reinforced the concept of environmental factors playing a role in the intensity of the Haitian cholera outbreak (Jutla et al., 2013). The whole genomic study undertaken by Katz et al. (2013) spurred further debate concerning Haitian cholera. They compared 23 Haitian *V. cholerae-*O1 genomes with 108 available *V. cholerae* genomes isolated from geographically distinct sources and concluded the Haitian isolates were nearly identical to Nepalese strains, consistent with the previous conclusion of Hendriksen et al. (2011). However, in contradiction to this conclusion, they also showed the Haiti isolates to be clearly distinguishable from isolates circulating elsewhere in the world. Katz et al. (2013) concluded the Haitian epidemic *V. cholerae* isolates were untransformable, with insertion of horizontally transmitted DNA was not detected. They did not examine *V. cholerae* non-O1 isolated in Haiti. However, the concept of a single source introduction of Haitian cholera from Nepal (Grad and Waldor, 2013) remains to be proven as the sole cause of all cholera and cholera-like diarrheal disease in Haiti in 2010.

Thus, even though whole genome analyses provided evidence for a major role of *V. cholerae* O1 in the Haiti epidemic, the issue is not settled. Mutreja et al. (2011) pointed to the cradle of seventh pandemic cholera by studying 136 WGSs of *V. cholerae* seventh pandemic El Tor isolates collected over the past 40 years, including 18 previously published genomes of *V. cholerae* El Tor and classical biotypes. They traced three independent overlapping waves of cholera descending from a 1950s ancestor isolated in the Bay of Bengal region and also suggested transcontinental outbreaks of cholera that appear to have been caused by genetically similar *V. cholerae*. This can be interpreted equally well, as evidence of the ubiquitous nature of this bacterium that has been shown to be autochthonous to the aquatic environment (Colwell, 1996).

## Conclusion

Technological advances, including automation of genomic fingerprinting and next generation sequencing have significantly improved cholera epidemiology. High-throughput sequencing has triggered output of an enormous amount of data. Bioinformatic tools are being developed that help to advance the molecular biology revolution. However, no single fingerprinting method is sufficient, as each method has its advantages and limitations. Nevertheless, a combination of methods can provide robust information and ensure resolution of epidemiological controversies.

Phenotypic fingerprinting continues to be useful for initial isolation, identification, and classification of *V. cholerae.* Molecular fingerprinting, i.e., VNTR and MLVA, can be useful in combination with WGS to achieve more precise discrimination in outbreaks caused by *V. cholerae* (Hasan et al., 2012) and become complementary to whole genome analyses. Although WGS promises to deliver highest resolution for a genomic-based epidemiology, differential annotation of whole genome data has generated intense debate exemplified by cholera in Haiti in 2010. Inter-laboratory comparative studies of WGS data no doubt will prove useful in reconciling the issues. A common platform for sharing whole genomic data is, therefore, strongly recommended. The newer generation of sequencing technologies has the potential to generate complete genomes in increasing frequency to allow precise genomic typing *in silico*, which will be complementary to the conventional typing strategies. Although cost of long read third generation sequencing is relatively high now, the cost is continuing to reduce to the point where it will soon be uniformly affordable. For now, it is concluded that combinations of phenotypic and molecular fingerprinting and WGS offer strategies for improved tracking, monitoring, and treating cholera.

## Conflict of Interest Statement

The authors declare that the research was conducted in the absence of any commercial or financial relationships that could be construed as a potential conflict of interest.

## Footnotes

1 http://highwire.stanford.edu/

2 http://www.ncbi.nlm.nih.gov/pubmed
